# A General Analysis of the Impact of Digitization in Microwave Correlation Radiometers

**DOI:** 10.3390/s110606066

**Published:** 2011-06-03

**Authors:** Xavier Bosch-Lluis, Isaac Ramos-Perez, Adriano Camps, Nereida Rodriguez-Alvarez, Enric Valencia, Hyuk Park

**Affiliations:** Remote Sensing Lab, Signal Theory and Communications Department, Building D3, Universitat Politècnica de Catalunya and IEEC CRAE/UPC, E-08034 Barcelona, Spain; E-Mails: isaacramos@tsc.upc.edu (I.R.-P.); camps@tsc.upc.edu (A.C.); nereida@tsc.upc.edu (N.R.-A.); valencia@tsc.upc.edu (E.V.); park.hyuk@tsc.upc.edu (H.P.)

**Keywords:** microwave, correlation, radiometers, digital, sampling, quantization

## Abstract

This study provides a general framework to analyze the effects on correlation radiometers of a generic quantization scheme and sampling process. It reviews, unifies and expands several previous works that focused on these effects separately. In addition, it provides a general theoretical background that allows analyzing any digitization scheme including any number of quantization levels, irregular quantization steps, gain compression, clipping, jitter and skew effects of the sampling period.

## 1. Introduction

Microwave radiometry is today a mature technology that was first used in radio-astronomy in the 1930s [1]. Since then, a large number of microwave radiometers have been developed for remote sensing applications to measure a wide range of natural phenomena (for examples, see [2–5]). Continuous technological evolution has given these systems new capabilities and features. One of the most relevant new technologies in the 1960s [6,7] was the digitization of the signals and the capabilities that emerge from specialized processing platforms such as today powerful an omnipresent Digital Signal Processors (DSP) and Field Programmable Gate Arrays (FPGA). Although digitization provides versatility, re-configurability and other advantages for microwave radiometry, it also has side effects that must be carefully analyzed.

Digitization effects can be separated into quantization and sampling effects. Mainly, the effects related with the quantization of the input signal (thermal noise) imply the loss of its statistical properties due to the non-linear quantization process. Consequently, it is not possible to apply the well-known Gaussian statistical relationships to the quantified signal. This effect has a large impact when limited quantization levels are considered, and it can be mitigated by increasing the number of quantization levels. Non-linear effects studies on Gaussian signals started with the early analysis of the spectrum of clipped noise by Van Vleck and Middleton [8]. In the late 1950s, Price published a work focusing on the relationship between the ideal correlation between two random signals with Gaussian probability density function (pdf), and the correlation measured after a non-linear manipulation of these random signals [9]. This relationship is now used to study the effects of arbitrary quantization schemes on the correlation of two signals.

Sampling has an impact on the correlation of the two input signals by creating spectral replicas. Additional noise can be added to the mean correlation value due to spectra replication depending on the ratio between the sampling frequency and the signal’s bandwidth. Moreover, the sampling period and its inaccuracies (skew and jitter in the sampling periods) of the Analog to Digital Converter (ADC) can affect and distort the sampled signal and so the cross-correlation value.

To compare different sampling and quantization schemes an equivalent integration time is defined as the one required to obtain the same resolution as in the ideal (analog) correlation. Hagen and Farley [10] conducted important work on this topic, where the effective time was defined and some easy-to-calculate digitization configurations were analyzed in depth.

More recent works have extended the initial digital radiometer proposed by Weinreb in 1961 [6] for auto-correlation spectrometers in the radio-astronomy field to include total power radiometers, polarimeters [11], and digitization impact on interferometric radiometers [12–15].

This work provides a general context to analyze the digitization effects on the cross-correlation of two Gaussian random signals in the most general case including any number of quantization levels, irregular quantization steps, gain compression, clipping, bandwidth, sampling frequency, and the skew and jitter inaccuracies of the sampling periods.

In Section 2 a general analysis of the correlation over non-linear functions applied to Gaussian noise is given. In Section 3 quantization effects are analyzed as a particular case of the results of the previous section. Section 4 analyses the sampling process. Section 5 evaluates the correlation variance due to the digitization. Finally, Section 6 presents the main conclusions of this study.

## 2. Non-Linearity Impact on the Correlation of Two Gaussian Random Signals

The input signal of a microwave radiometer comes from natural thermal emission. Let us consider here two independent Gaussian random processes with a certain cross-correlation coefficient characterized by their probability density function (pdf). Furthermore, both input signals are assumed to be statistically identical and stationary, so the properties of the signals at a given time (T_0_) are independent of T_0_. Furthermore, both signals fulfill that their statistical properties can be deduced from a single, sufficiently long sample of the process (realization), *i.e.*, they are ergodic processes [16]. Thus, the variance of each series is constant and the covariance between elements depends only on their time separation. Finally, let *x* and *y* be input signals of a radiometer fulfilling the previous requirements. Usually, these random variables have zero mean (μ*_x_*_,_*_y_* = 0), except for offsets, and are considered to have a standard deviation different from one . In this case, the joint probability of density is defined as in Equation (1):

(1) $$f(x,y)=\frac{1}{2{\pi \sigma }_{x}\sigma_{y}\sqrt{1-\rho_{xy}^{2}}}{\text{e}}^{-\frac{1(x^{2}+y^{2}-2\rho_{xy}^{2}xy)}{2\;\;\;\;\;\;\;\;\sigma_{x}\sigma_{y}(1-\rho_{xy}^{2})}}$$

where:

- σ*_x,y_* are the standard deviation of *x* and *y* signals, and
- *ρ_xy_* is the Pearson correlation coefficient and it is defined as the ratio between the covariance of *x* and *y* and the geometric mean of the product of their variances (Equation (2)):
  (2) $$\rho_{xy}=\frac{\langle xy*\rangle }{\sqrt{\langle xx*\rangle \langle yy*\rangle }}$$

where the brackets 〈·〉 indicate a statistical average, over a time sequence with identical statistics (ergodic properties). Price’s theorem [9] relates the correlation coefficient of *x* and *y* after (*R_xy_*) and before (*ρ_xy_*) the non-linear functions (*g*_1_(·) and *g*_2_(·)) to the random variables (Equation (3)):

(3) $$\frac{\partial^{k}R_{xy}}{\partial \;\rho_{xy}^{k}}=\sigma_{x}^{k}\sigma_{y}^{k}\langle g_{1}^{k}(x)g_{2}^{k}(y)\rangle$$

where:

- *R_xy_* is the correlation coefficient of *x* and *y* after the non-linear functions *g*_1_(·) and *g*_2_(·) are applied to *x* and *y*, respectively
- *ρ_xy_* is the ideal correlation coefficient of *x* and *y* (before the non-linear functions *g*_1_(·) and *g*_2_(·))
- $\sigma_{x,y}^{k}$ are the standard deviation of *x* and *y* signals, (for the sake of clarity, from now on $\sigma_{x,y}^{k}\;=\;1$ ), and
- *k* denotes the *k^th^* derivative of each function.

From Equation (3), it is possible to retrieve *ρ_xy_*, the ideal correlation between *x* and *y*, by *k*-derivation and further correlating the *g*_1_(*x*) and *g*_2_(*y*) signals and then *k*-integrating with respect to the *ρ_xy_* variable from −1 to +1. These operations can be defined as a relationship between the ideal and the non-linear correlations. It is defined here as the function *q* [·]:

(4) $$\rho_{xy}=q^{-1}[R_{xy}]$$

Considering the correlation of two signals at a delay different form zero (*x* → *x*(t = 0) and *y* → *y*(t = *τ*)) the effect of the hardware response can be included as depending on *τ*, then Equation (2) becomes:

(5) $$\rho_{xy}(\tau )=\langle xy*\rangle =\rho_{xy}(0){\tilde{r}}_{xy}(\tau )$$

where:

- *ρ_xy_*(0) is the Pearson correlation coefficient (Equation (2)) at *τ* = 0, and
- ${\tilde{\text{r}}}_{xy}(\text{t})≜\frac{{\text{e}}^{-\text{j}2\pi f_{0}\text{t}}}{\sqrt{{\text{B}}_{x}{\text{B}}_{y}}}\int_{0}^{\infty }{\text{H}}_{x}(f)\;{\text{H}}_{y}^{*}(f){\text{e}}^{\text{j}2\pi f\text{t}}df$ is the so-called Fringe-Washing Function (FWF), as defined in [17], where B*_x_* and B*_y_* are the equivalent noise bandwidths, *f_0_* is the central frequency, and H*_x_*(*f*) and H*_y_*(*f*) are the normalized frequency responses of the *x* and *y* channels, respectively. Note that, if the channels’ frequency response are equal, with rectangular shape of bandwidth B, and centered frequency *f_0_*, the fringe-washing function reduces to the well-known formula r̃*_xy_*(*τ*) = sinc(B*_xy_τ*).

Equation (5) depends on the correlation coefficient and the hardware features. Usually, to avoid an underestimation of the correlation coefficient, Equation (4) has to be compensated by the existing delay between *x* and *y* as it follows:

(6) $$\rho_{xy}(0)=\frac{q^{-1}[R_{xy}(\tau )]}{{\tilde{\text{r}}}_{xy}(\tau )}$$

Assuming that the *x* and *y* are stationary random processes (thermal noise radiation) that fulfill the ergodic property, then Equations (2) and (5) can be calculated in practice using the cross-correlation technique (multiplication and time averaging).

## 3. Quantization Impact

This section particularizes the previous analysis to a generic ADC function. The degree of non-linearity of an ADC function depends on the number of quantization levels and the ADC span window (V_ADC_ = X*_M_* − X*_o_*), going from 2 levels (1 bit) the most non-linear scheme, up to an infinite number of levels with an infinite V_ADC_, which can be considered linear. As explained before, quantization does not take into account the sampling, so that this analysis considers an infinite frequency sampling (*F_s_* → ∞). Sampling effects will be added in Section 5.

**Figure 1.**
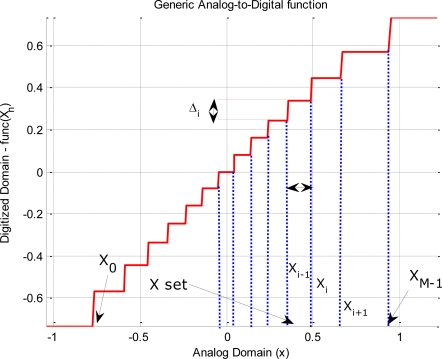
Generic analog-to-digital transfer function. The function plotted has compression gain and different steps in analog and digitized domains.

Equation (7) shows a general quantization function (Figure 1):

(7) $$g_{i}(x)=\sum_{m=0}^{M-1}\Delta_{m}^{i}\;u(x-{\text{X}}_{m})$$

where:

- *x* is the value of the signal in the analog domain (continuous in time and values),
- *X_m_* and X*_m_*_+1_ are two consecutive threshold values of the input signal in the X̄ space (see Figure 1), where X̄ is the set of all the input threshold values in the digital domain (X̄; = {X*_m_*}, *m* = 0… *M* − 1). Note that, in the most general case the distance between two consecutive steps does not have to be constant (X*_m_* − X*_m_*_−1_ ≠ X*_m_*_+1_ − X*_m_*),
- *u*(*x* − X*_m_*), is the Heaviside function (step function) centered at X*_m_*,
- $\Delta_{m}^{i}$ is the quantization step value between the (X*_m_*_−1_, X*_m_*) interval. Note that, in the most general case the distance between two consecutive steps does not have to be constant either ( $\Delta_{m}^{i}\neq \Delta_{m+1}^{i}$), and
- the values X_0_ and X*_M_*_−1_ define the lower and upper bounds of the quantization window. Again, in a general case, the lower bound can be different from the upper bound (X*_m_* ≠ −X*_M_*_−1_).

The first derivative function of Equation (7) is given by:

(8) $$\frac{\partial g_{i}(x)}{\partial x}=\sum_{m=0}^{M-1}\Delta_{m}^{i}\delta (x-{\text{X}}_{m})$$

where *δ* is the Kronecker’s delta: *δ* = 1 if *x* = X*_m_* and *δ* = 0 if *x* ≠ X*_m_*.

Substituting the results obtained in Equation (8) in Equation (3), with different *g* (·) functions for *x* and *y*, and considering the first derivative function (*k* = 1), the relationship between the correlation of two signals before and after the quantization process is obtained in Equation (9):

(9) $$\frac{\partial R_{xy}}{\partial \rho_{xy}}=\frac{1}{2\pi \sqrt{1-\rho_{xy}^{2}}}\overset{\infty }{\underset{-\infty }{\int \int }}\sum_{p=0}^{P-1}\Delta_{p}^{x}\delta (x-{\text{X}}_{p})\sum_{q=0}^{Q-1}\Delta_{q}^{y}\delta (y-{\text{Y}}_{q}){\text{e}}^{-\frac{1(x^{2}+y^{2}+2xy\rho_{xy})}{\;\;\;\;\;\;\;\;\;\;2\;\;(1-\rho_{xy}^{2})\;\;\;\;\;\;\;\;\;\;\;\;\;\;\;\;\;\;\;\;\;\;\;\;\;\;\;\;\;\;\;}}dxdy$$

Thereafter, it is easy to compute the integrals over the *x* and *y* domains by evaluating them at the points where Kronecker’s delta does not vanish. Therefore, Equation (9) becomes Equation (10):

(10) $$\frac{\partial R_{xy}}{\partial \rho_{xy}}=\frac{1}{2\pi \sqrt{1-\rho_{xy}^{2}}}\sum_{p=0}^{P-1}\sum_{q=0}^{Q-1}\Delta_{p}^{x}\Delta_{q}^{y}{\text{e}}^{-\frac{1({\text{X}}_{p}^{2}+{\text{Y}}_{q}^{2}+2{\text{X}}_{p}{\text{Y}}_{q}\rho_{xy})}{2\;\;\;\;\;\;\;\;\;\;\;\;\;\;\;\;\;\;\;\;\;\;\;\;\;\;\;\;\;\;\;\;\;\;\;(1-\rho_{xy}^{2})}}$$

and finally, the integration over the *ρ_xy_* domain is performed from −1 to 1.

(11) $$R_{xy}=\int_{-1}^{1}\frac{1}{2\pi \sqrt{1-\rho_{xy}^{2}}}\sum_{p=0}^{P-1}\sum_{q=0}^{Q-1}\Delta_{p}^{x}\Delta_{q}^{y}{\text{e}}^{-\frac{1(X_{p}^{2}+Y_{q}^{2}+2X_{p}Y_{q}\rho_{xy})}{2\;\;\;\;\;\;\;\;\;\;\;\;\;\;\;\;\;\;\;\;\;\;\;\;\;\;\;\;\;\;\;\;\;(1-\rho_{xy}^{2})}}d\rho_{xy}$$

From Equation (11) it is straightforward to include the impulse response of the system by considering several values of *ρ_xy_* for several delays (τ):

(12) $$R_{xy}(\tau )=\int_{0}^{\rho_{xy}(\tau )}\frac{1}{2\pi \sqrt{1-\rho_{xy}^{2}(\tau )}}\sum_{p=0}^{P-1}\sum_{q=0}^{Q-1}\Delta_{p}^{x}\Delta_{q}^{y}{\text{e}}^{-\frac{1({\text{X}}_{p}^{2}+{\text{Y}}_{q}^{2}+2{\text{X}}_{p}{\text{Y}}_{q}\rho_{xy}(\tau ))}{2\;\;\;\;(1-\rho_{xy}^{2}(\tau ))}}d\rho_{xy}(\tau )$$

In some particular and simplified cases, Equation (12) can be obtained analytically, for instance in the case of quantifying with two levels (one bit, *Q* = P = 1 and $\Delta_{0}^{x}=\Delta_{0}^{y}=2$). In this case, Equation (12) becomes the well-known solution stated in Equation (13) [9]:

(13) $$R_{xy}\left(\rho_{xy}(\tau )\right)=\frac{2}{\pi }\text{arcsin}\;(\rho_{xy}(\tau ))$$

Otherwise, when the quantization scheme is more complex Equation (12) can only be analyzed numerically, such as having different numbers of bits, when signals are clipped, when the quantization steps are irregular, when the ADC has a non-linear circuit before that exhibits gain compression, when there is no symmetry between the positive and negative parts or many other possibilities.

**Figure 2.**
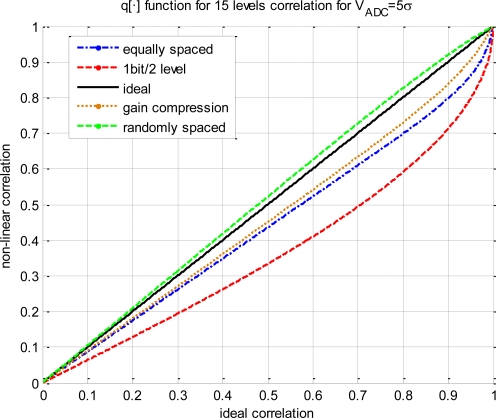
Relationship between the non-linear and the ideal correlation for different digitization schemes.

Figure 2 has been obtained from Equation (12) for several quantization schemes. Two *q*[·] functions are plotted for reference, the coarsest quantization scheme (*i.e.*, 2 levels/1 bit, Equation (13)), and the ideal one (*i.e.*, infinite levels, R*_xy_*(*τ*) = *ρ_xy_*(*τ*)). Three more functions are shown considering an ADC span window of 5*σ_x,y_* (V_ADC_ = 5*σ_x,y_*), 15 quantization levels or 3.9 bits (log_2_15 = 3.9, note that it is not necessary to have an integer number of bits), but changing the non-linear function. Several *g*(*x*) functions have been considered such as equally spaced levels, using gain compression modeled by an hyperbolic tangent (*g*(*x*) = tan (*x*)) or having randomly spaced levels. All results are different from the others, showing the need of a deeper analysis.

The root mean square error (RMSE) coefficient between R*_xy_*(*τ*) and *ρ_xy_*(*τ*) has been chosen to measure the degree of linearity of *q*[·]. So, the RMSE has been selected to be the figure of merit for the comparison of several quantization schemes. Obviously, the linearity of any *q*[·] function is better as the RMSE is closer to zero. For a given application, there is a maximum distortion of the RMSE that can be afforded by the *q*[·], and that is called Max_RMSE_. If the relationship between the quantized and the ideal correlations is linear enough (RMSE < Max_RMSE_), then the value of the quantized correlation matches with the ideal correlation and there is no need of using the *q*^−1^[·] function. Otherwise, if it is not linear enough (RMSE ≥ Max_RMSE_) then, the obtained correlation has to be modified by the *q*^−1^[·] function in order to retrieve the real correlation.

**Figure 3.**
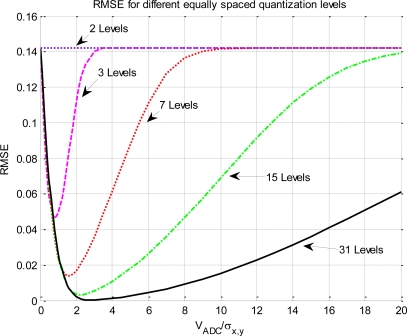
Root mean square error and ADC span window relationship for different equally spaced quantification levels.

Figure 3 presents the RMSE computed using Equation (12), with different quantization levels and V_ADC_. Each curve has a minimum that corresponds to the optimum configuration of V_ADC_ with respect *σ_x,y_*. As the number of quantization levels increases, the minimum value of RMSE curve gets closer to 0, and the V_ADC_/*σ_x,y_* range where the curves remain close to 0 increases as well, since the function *q*[·] is linear over a wider range of input powers. By inspecting Figure 3 it is clear that for V_ADC_ < 2*σ_x,y_*, clipping has a dominant impact over the non-linear correlation. This effect has the maximum impact when the clipping reduces the whole ADC to a 2 level decision (1 bit). As the clipping effect increases, the RMSE value converges to the RMSE of the 1 bit/2 levels quantization (RMSE = 14.2%), which is the most non-linear quantization scheme possible.

On the other hand, plots also have a common trend in the region above the minimum, where the ratio V_ADC_/*σ_x,y_* increases. As the number of quantization levels increases, the ideal and non-linear relationship (*q*[·]) becomes more linear over a wider range of input power (the region around the minimum widens). But when V_ADC_/*σ_x,y_* → ∞, the RMSE asymptotically increases again up 14.2% (the value of the 1 bit/2 levels) for all the quantization schemes. This effect can be understood as fewer bits are being used as the V_ADC_/*σ_x,y_* ratio increases, and it can be understood as a decreasing of the effective number of bits. The maximum effect occurs when the V_ADC_ is so spread over *σ_x,y_* that only two levels are effectively used for quantization.

Another conclusion that can be drawn from Figure 3, it is that each quantization level has its own optimum ADC span window (V_ADC_) with relation of the standard deviation of the input signal. This is a critical design parameter and it has been summarized on Table 1 for some quantization schemes.

**Figure 4.**
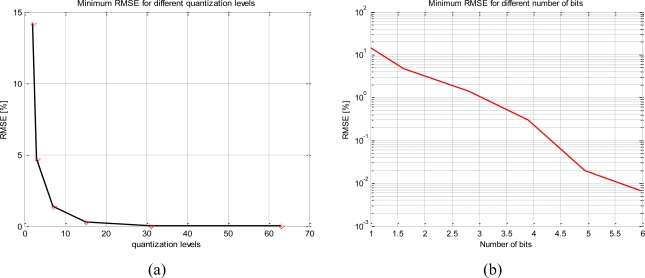
Minimum root mean square errors for different quantization levels, it decreases following an exponential trend, **(a)** RMSE decreasing *vs*. the quantization levels, and **(b)** RMSE in a semi-log axis decreasing *vs*. the number of bits.

Table 1 shows that the two-level quantization scheme is not sensitive to the V_ADC_/*σ_x,y_* relationship and the RSME is always 14.2% and, on the other hand for 31 levels the relationship between quantized and ideal correlation is almost linear. Furthermore, as the number of levels increases the minimum RMSE decreases exponentially (Figure 4) and it occurs at a higher V_ADC_/*σ_x,y_* ratio.

**Figure 5.**
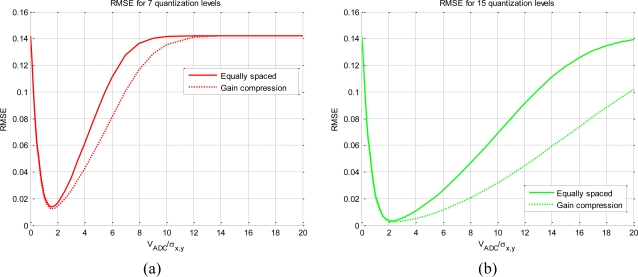
Root mean square error for different levels and different spaced quantification levels, **(a)** 7 quantization levels, and **(b)** 15 quantization levels.

Figure 5 shows the effect of using various *g*(*x*) functions. It compares an equally spaced quantification with a hyperbolic tangent spacing, used to compress the gain.

In both cases, Figure 5(a) (seven levels) and Figure 5(b) (15 levels), there are no changes in the clipping area, but there are as the V_ADC_/σ*_x,y_* ratio increases. The effect is that an ADC with gain compression has lower RMSE values than the same ADC but with equally spaced level scheme for a wider area. This is equivalent to increasing the number of quantization levels. An ADC with gain compression can be useful to ensure linearity over a wide range of input signal power, reducing the necessary number of bits. A common radiometric application where a high range of input power is measured is during the radiometric T_hot_ − T_cold_ calibration.

## 4. Impact of Quantization on the Correlation Spectrum

In [7] some analytically easy-to-solve quantization schemes were analyzed using Taylor series approximations when *ρ_xy_* ≪ 1, which applies in most radio-astronomical measurements, but not necessarily in Earth remote sensing or during a radiometric calibration. The cross-correlation spectrum, the frequency power density is spread and distorted due to quantization step and clipping. In this work, the spectrum is computed following Equation (12). Therefore, the analysis can be exhaustive and include different quantization levels for two input signals for any complexity, gain compression, different bandwidths, and different channel frequency responses. The frequency power density is the Fourier transformation of the cross-correlation function, which is the Wiener-Khinchin theorem (Equation (14)):

(14) $$S_{xy}(f)=\int_{-\infty }^{\infty }R_{xy}(\tau ){\text{e}}^{-\text{j}2\pi \tau f}d\tau =F\{g_{1}(x(t))\}F{\left\{g_{2}(x(t))\right\}}^{*}$$

Furthermore, Equation (14) also shows the relationship of the cross-correlation spectrum with the individual signals spectrum, which is the multiplication of the Fourier transforms of the output signals of the quantization functions. By replacing Equation (4) in Equation (14) the spectral power density of the non-linear cross-correlation can be readily obtained (Equation (15)):

(15) $$S_{xy}(f)=\int_{-\infty }^{\infty }q[\rho_{xy}(\tau )]{\text{e}}^{-\text{j}2\pi \tau f}d\tau$$

where the function *q*[*ρ_xy_*(*τ*)] can be obtained analytically (following Equation (13)) or numerically (following Equation (12)).

**Figure 6.**
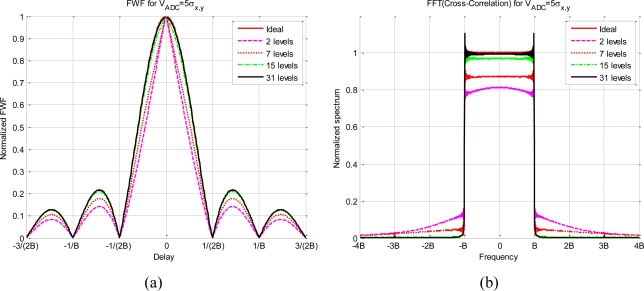
Effect of the quantization on the cross-correlation spectrum, **(a)** ideal cross-correlation (*ρ_xy_*(*τ*)) and cross-correlation for different quantization levels, and **(b)** their corresponding spectrum.

The effect of the quantization in the cross-correlation spectrum is to decrease and distort it in the pass-band and spread it over the rejected band. Figure 6 presents an example of the impact of the quantization on the spectrum computed following Equation (15). The input signals, *x* and *y*, that have been considered for this analysis have a rectangular frequency channel’s response with a pass-band bandwidth of 2B and a V_ADC_ = 5*σ_x,y_* relationship for all the quantization schemes.

Figure 6(a) presents the *x* and *y* linear cross-correlation on the delay domain for several quantization levels. The lower the number of levels, the higher the correlation distortion. Figure 6(b) shows the corresponding spectrum of cross-correlations presented in Figure 6(a), which have been computed following Equation (15). As expected, the two-level scheme has the highest spectrum distortion, the spectrum spread increases on the rejected band and the pass-band decreases.

**Figure 7.**
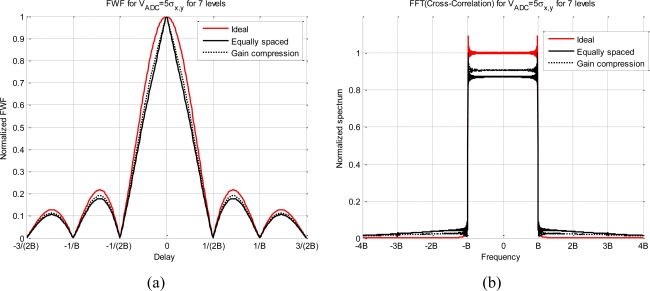
Effect of the quantization on the cross-correlation spectrum, **(a)** ideal cross-correlation (*ρ_xy_*(*τ*)) and cross-correlation for two different quantization schemes using 7 levels, and **(b)** presents their corresponding spectra.

Table 2 summarizes these results, the main conclusion is that using V_ADC_ = 5*σ_x,y_* and at least 15 quantization levels it can be assumed that it neither spreads nor distorts the spectrum. Figure 7 shows the effect for two *g*(*x*) functions and for seven quantization levels. It can be observed that the compression gain reduces the distortion and the spread spectrum.

In this section, it has been shown that the quantization can be successfully expressed and analyzed as a non-linear transformation. Despite the quantization can affect significantly the value of the cross-correlation, it is possible to recover the ideal correlation using the inverse function *q*^−1^[·], which can be obtained numerically. Furthermore, for each quantization scheme there is an optimum configuration, in terms of linearity, of the V_ADC_/*σ_x,y_* relationship. Furthermore, quantization distorts and spreads the cross-correlation spectrum. Finally, the quantization not only has an impact on the value of the cross-correlation and on the spread of the spectrum, but it increases the standard deviation of the cross-correlation, as it will be shown in the next section.

## 5. Sampling Impact

The main effect of sampling is that it creates replicas of the spectrum. Usually, in a standard digital radiometer topology there is an anti-aliasing filter before the digitization process. Even in this case, the digitization can introduce aliasing due to the spread of the quantized spectrum, which depends on the sampling frequency (*F_s_*), and the baseband bandwidth (B). To understand how this effect impacts on the sampling it is necessary to define the Signal to Noise Ratio (SNR) as in Equation (16):

(16) $$\text{SNR}=\frac{{\bar{R}}_{xy}}{\sigma_{R}{}_{{}_{xy}}}$$

where *R̄_xy_* is the expected non-linear cross-correlation value, and *σ_R_xy__* is the standard deviation.

Moreover, the SNR term is related with the radiometric resolution [18], which is one of the figures of merit of any radiometer. The SNR can decrease due to an increase of the standard deviation of the correlation or due to a decrease of the *R̄_xy_*, *i.e.,* a noise increment on the pass-band (Equation (2)).

**Figure 8.**
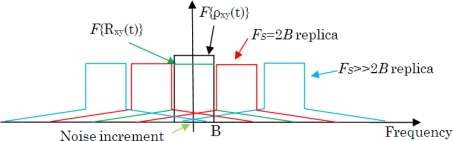
Effect of the sampling on the correlation spectrum, (*F*{·} stands for Fourier transform). Spectrum of the ideal and the quantized correlation at baseband, black and green, respectively. In red, it is the first spectrum replica for the minimum sampling frequency which fulfills the Nyquist criterion (*F*s = 2B). In blue, it is the first spectrum replica for *F*s ≫ 2B.

Figure 8 shows an ideal baseband spectrum with an infinite sampling frequency (*F*{*ρ_xy_*(*τ*)}, in black) and a cross-correlation spectrum spread due to quantization (*F*{*R_xy_*(*τ*)}, in green) for different *F_s_* and its replicas [19,20]. After quantization and sampling, and further cross-correlation of the signals, the quantization tail of the Nyquist’s replica (*F_s_* = 2B, in red) overlaps the pass-band of the baseband replica, so an extra amount of noise is included in the measurement of *R̄_xy_*, and the effect is a decrease of the calculated mean value (Equation (2)). On the other hand, the standard deviation remains constant, but the SNR decreases, which has a large impact when considering sub-Nyquist sampling.

If the sampling frequency increases, then the first replicas (*F_s_* ≫ 2B, in blue) appear further away from the band-pass replica and the tail effect has less impact than before. This is why when only few quantization levels are used an increase of the sampling frequency has a significant impact on the radiometric resolution. Even though, it has less impact when more quantization levels are used.

**Figure 9.**
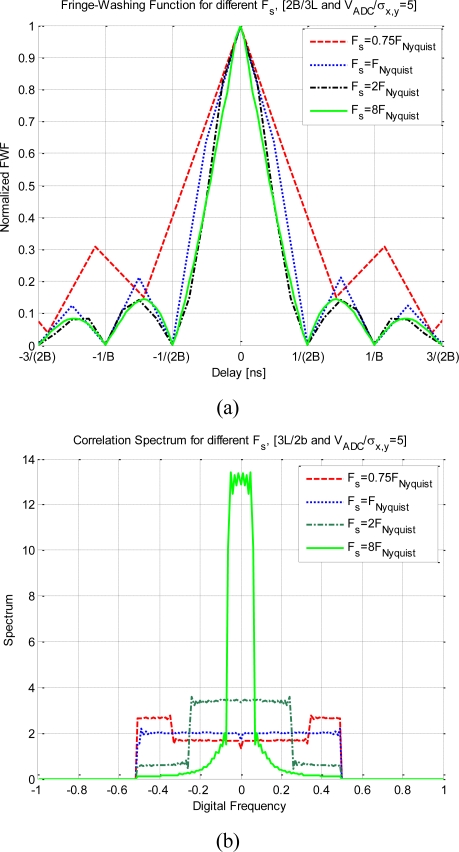
Impact on the quantized correlation of different sampling frequencies. For 2 quantization levels, a bandwidth 2B (assuming a band-pass signal), and V_ADC_ = 5*σ_x,y_*. **(a)** shows the fringe-washing function deformation, and **(b)** shows the effect on their spectrum.

Figure 9 shows the impact on the quantized correlation of different sampling frequencies. The considerations for these plots are a 2 quantization levels scheme, a noise equivalent band-pass bandwidth of 2B (assuming a band-pass signal), and V_ADC_ = 5*σ_x,y_*, taking into account the previous considerations about the spectrum replicas. Figure 9(a) shows the deformation of the fringe washing function for a set of sampling rates. The sub-Nyquist sampling (*F_s_* = 0.75 F_Nyquist_) has the largest deformation: it is wider, sharper than the rest of them, and the side lobes are higher.

For the exactly Nyquist sampling rate the fringe-washing function is still sharp and wide. For a higher sampling rate (*F*_s_ > 2 F_Nyquist_) the function is not affected by the sampling rate. On the other hand, the spectrum in Figure 9(b) is totally distorted in the sub Nyquist sampling rate case, and recovers its original shape as the sampling rate increases.

**Figure 10.**
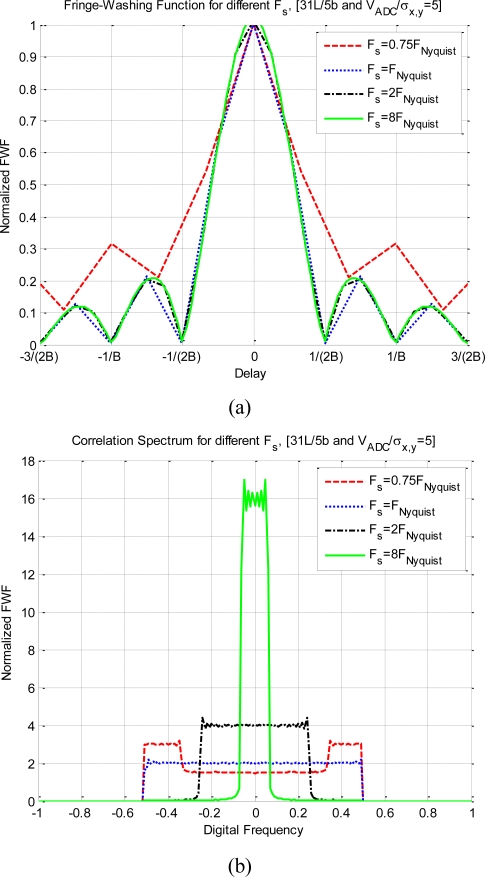
Impact on the quantized correlation of different sampling frequencies. For 31 quantization levels, a bandwidth 2B, and V_ADC_ = 5*σ_x,y_*. **(a)** shows the deformation for the fringe-washing function, and **(b)** shows the effect on their spectrum.

Figure 10 shows the impact on the quantized correlation of different sampling frequencies. The difference from the previous analysis is that in these plots 31 quantization levels have been considered. As this quantization scheme spread less the spectrum than the previous one, a better performance is achieved for lower sampling rates. Figure 10(a) shows the distortion of the fringe-washing function considering different sampling rates. The sub-Nyquist sampling (*F_s_* = 0.75 F_Nyquist_) has the largest distortion: it is wider, sharper than the rest of them, and the side lobes are higher. For a larger sampling rate (*F*_s_ ≥ F_Nyquist_) the function is not affected by the sampling rate. On the other hand, in the Figure 10(b), the spectrum is completely distorted for the sub-Nyquist sampling rate case, and recovers its original shape as the sampling rate increases. As expected, it has better performance for higher sampling rates.

## 6. Sampling Rate Inaccuracies

In the conversion from analog to digital signals, the sampling frequency is normally assumed to be constant. Nevertheless, the sampling clock is prone to interferences, thermal drifts and other effects that can change the oscillating frequency and introduce sampling inaccuracies.

**Figure 11.**
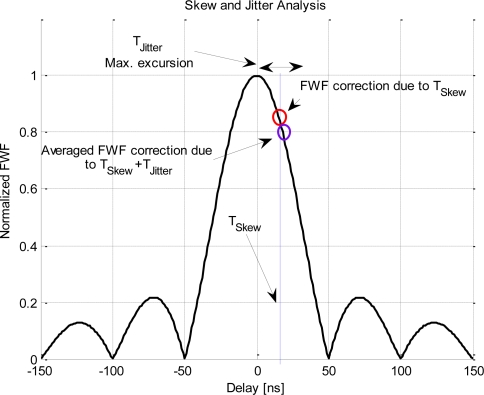
Impact of the clock inaccuracies on the correlation value. T_skew_ is the sampling rate offset between *x* and *y*, and T_Jitter_ is its fluctuations due to clock inaccuracies.

Figure 11 shows the effect of these inaccuracies, that can be summarized into skew (T_Skew_), which is the delay offset between the sampling time of *x* and *y*, and jitter, which is the time fluctuation above the mean value (T_Jitter_). The impact of the skew is changing the point where the FWF is evaluated and a phase rotation of the correlation coefficient if the working frequency is not exactly zero. Meanwhile, jitter creates a perturbation for each correlation sample around the mean point with zero mean.

A clock skew error not only has an effect on the modulus of the correlation coefficient, but it affects its phase by rotating the real and imaginary parts [21], as it is shown in Equation (17):

(17) $$\begin{array}{l} \langle x(n{\text{T}}_{1})y^{*}(n{\text{T}}_{2})\rangle \\ \;\;\;\;\;\;\;\;\;\;\;\;\;\;\;\;\;\;\;\;\;\;\;\;\;\;\;\;\;\;\;\;\;\;=r_{xy}({\text{T}}_{1}-{\text{T}}_{2})\{[\rho_{R}\text{cos}\omega_{\text{r}}({\text{T}}_{1}-{\text{T}}_{2})-\rho_{I}\text{sin}\omega_{\text{r}}({\text{T}}_{1}-{\text{T}}_{2})]\\ \;\;\;\;\;\;\;\;\;\;\;\;\;\;\;\;\;\;\;\;\;\;\;\;\;\;\;\;\;\;\;\;\;\;+j[\rho_{I}\text{cos}\omega_{\text{r}}({\text{T}}_{1}-{\text{T}}_{2})+\rho_{R}\text{sin}\omega_{\text{r}}({\text{T}}_{1}-{\text{T}}_{2})]\} \end{array}$$

where:

- 〈*x*(*x*T_1_)y*(*n*T_2_)〉 = *ρ* = *ρ_R_* + *jρ_1_*, the correlation coefficient is a complex value with modulus and phase ( $\text{j}=\sqrt{-1}$)
- $\omega_{\text{r}}=2\pi \frac{f_{r}}{F_{s}}$ is the ratio between the residual frequency (*f_r_*) and the sampling frequency, residual digital frequency of the signals, and *F_s_* = 1/T*_s_*
- *r_xy_*(T_1_ − T_2_) is the fringe-washing function evaluated at T_1_ − T_2_, and
- T_1_ and T_2_ are the sampling periods for the *x* and *y* inputs, respectively. Both sampling times are identical except for the skew and jitter effects ((T_1_ − T_2_) = T_Skew_ + T_Jitter_(*t*)).

On the other hand, the jitter is a random variable effect that can be statistically modeled as a polynomial of standard deviation depending on time ( $\sigma_{\text{Jitter}}=\sigma_{\text{app}}+\sqrt{c_{\mathrm{clk}}(n-m){\text{T}}_{s}}$), where *n* and *m* are two different samples. So that, it can be associated to two different and independent processes ([22,23]):

- the aperture jitter, which is related to the random sampling time variations in the ADC caused by thermal noise in the sample&hold circuit. The aperture jitter is commonly modeled as an independent Gaussian variable with zero mean and a standard deviation of *σ*_Jitter_ = *σ*_app_, and
- the clock jitter, which is a parameter of the clock generator that drives the ADC with the clock signal. The clock jitter is modeled as a Wiener process, *i.e*., a continuous-time non-stationary random process with independent Gaussian increments with a standard deviation equal to $\sigma_{\text{Jitter}}=\sqrt{c_{\mathrm{clk}}(n-m){\text{T}}_{s}}$.

Taking into account a wideband signal in jitter terms (fulfill the inequality 2*π*B*σ*_Jitter_ > 1). From here, *x* can be defined using its discrete-time Fourier transformation (Equation (18)):

(18) $$x(n{\text{T}}_{\text{s}}+{\text{T}}_{Jn})={\text{T}}_{\text{s}}\int_{-F_{\text{s}}/2}^{F_{\text{s}}/2}X(f){\text{e}}^{-\text{j}2\pi f(n{\text{T}}_{\text{s}}+{\text{T}}_{Jn})}\text{d}f$$

where T*_J_n__* is the value of the instantaneous jitter. Thence, following Equation (18) the correlation can be defined again using Equations (14) and (18):

(19) $$R_{xy}(\tau )=\langle x(m{\text{T}}_{\text{s}})y*(n{\text{T}}_{\text{s}}+{\text{T}}_{J_{{}_{\left|m-n\right|}}})\rangle =\langle \int_{-F_{\text{s}}/2}^{F_{\text{s}}/2}\left(X(f)Y^{*}(f){\text{e}}^{\text{j}2\pi f{\text{T}}_{J_{{}_{\left|m-n\right|}}}}\right){\text{e}}^{-\text{j}2\pi f(m-n){\text{T}}_{\text{s}}}\text{d}f\rangle$$

(20) $$R_{xy}(\tau )=\int_{-F_{\text{s}}/2}^{F_{\text{s}}/2}S_{xy}(f)\langle {\text{e}}^{\text{j}2\pi f{\text{T}}_{J_{\left|m-n\right|}}}\rangle {\text{e}}^{-\text{j}2\pi f(m-n){\text{T}}_{s}}\text{d}f$$

where:

- 〈e^j2π*f*T_*J*__|m−n|_^〉 is the expected value of the jitter and it depends on the jitter type [23],
- T*_J_*__|_*_m_*_−_*_n_*_|__ is the existing jitter between the *m^th^* sample of *x* and the *n^th^* sample *y*, and
- *τ* is the digital delay between the cross-correlation samples (*τ* = (*m* − *n*)T_s_).

Equation (21) applies for an aperture jitter and Equation (22) applies for the clock jitter:

(21) $$\langle {\text{e}}^{\text{j}2\pi f{\text{T}}_{Japp_{n}}}\rangle ={\text{e}}^{2\pi^{2}f^{2}\sigma_{\mathrm{app}}^{2}},\text{ and }\langle e^{\text{j}2\pi f{\text{T}}_{Japp_{{}_{\left|m-n\right|}}}}\rangle ={\text{e}}^{-2\pi^{2}f^{2}2\sigma_{\mathrm{app}}^{2}},\;\;\text{if n}\neq \text{m}\;\text{else}\;1$$

(22) $$\langle {\text{e}}^{\text{j2}\pi f{\text{T}}_{J{\text{clk}}_{n}}}\rangle ={\text{e}}^{2\pi^{2}f^{2}c_{\mathrm{clk}}{\text{nT}}_{\text{s}}},\text{ and }\;\;\;\langle {\text{e}}^{\text{j}2\pi f{\text{T}}_{{Jclk}_{\left|m-n\right|}}}\rangle ={\text{e}}^{-2\pi^{2}f^{2}c_{\mathrm{clk}}{\text{T}}_{\text{s}}|m-n|}$$

where *c_clk_* is the phase noise constant of the oscillator. Thence, the impact of the aperture jitter to the cross-correlation coefficient is shown in Equation (24):

(23) $$\begin{array}{l} {\overset{⌣}{R}}_{xy}(\tau )=\int_{-\infty }^{\infty }S_{xy}(f){\text{e}}^{-2\pi^{2}f^{2}\sigma_{\text{Jitter}}^{2}}{\text{e}}^{-\text{j}2\pi f\tau }df\\ \;\;\;\;\;\;\;\;\;\;\;\;\;\;=\int_{-\infty }^{\infty }S_{xy}(f){\text{e}}^{-2\pi^{2}f^{2}((2-\delta_{x})\sigma_{\mathrm{app}}^{2}+c_{\mathrm{clk}}{\text{T}}_{\text{s}}|m-n|)}{\text{e}}^{-\text{j}2\pi f\tau }\text{d}f \end{array}$$

(24) $${\overset{⌣}{R}}_{xy}(\tau )=R_{xy}(\tau )*\frac{e^{-\frac{\tau^{2}}{2\sigma_{\mathrm{app}}^{2}}}}{\sqrt{2\pi \sigma_{\mathrm{app}}}}*\frac{e^{-\frac{\tau }{2c_{\mathrm{clk}}}}}{\sqrt{2\pi c_{\mathrm{clk}}\tau }}$$

where:

- *R̆_xy_*(*τ*) is the cross-correlation coefficient modified by the jitter,
- *R_xy_*(*τ*) is the cross-correlation coefficient, and
* denotes the convolution operator,

From Equation (22) it can be inferred that the aperture jitter does not depend on the sampling rate and it has a low-pass filter effect on the correlation spectrum with its cut-off frequency depending on *σ*_app_. Particularizing Equation (24) in the case of having a rectangular spectrum, then Equation (24) becomes Equation (25), and the cross-correlation function is multiplied by an erf(*x*) function ( $\text{erf}(x)=\frac{2}{\sqrt{\pi }}\int_{0}^{x}e^{-t^{2}}\mathrm{dt}$):

(25) $${\overset{⌣}{R}}_{xy}(\tau )=\int_{-\text{B}/2}^{\text{B}/2}\sigma_{x}\sigma_{y}\rho (\tau ){\text{e}}^{-2\pi^{2}f^{2}(2-\delta_{x})\sigma_{\mathrm{app}}^{2}}{\text{e}}^{-\text{j}2\pi f\tau }\text{d}f=R_{xy}(\tau )\frac{\text{erf}\left(\frac{\pi }{\sqrt{2}}\text{B}\sigma_{\mathrm{app}}\right)}{\sqrt{2\pi \sigma_{\mathrm{app}}}}$$

As it can be seen, in the case of aperture jitter the coherence loss ratio (Equation (20)) is independent of the number of sampling points *N* (therefore of the integration block length T*_s_N*), while in the case of clock jitter it strongly depends on it (Equation (21)).

## 7. Analysis of the Cross-Correlation Variance due to the Digitization

In the previous sections, quantization and sampling have been analyzed in terms of the impact on the retrieved value of the correlation. In this section, the variances are analyzed taking into account both effects (quantization and sampling). In a general case, the output of a correlator of two quantized and sampled signals after averaging *N_q_* samples is defined as:

(26) $$R_{xy}(\tau )=\frac{1}{N_{q}}\sum_{n=1}^{N_{q}}g_{1}(x(n{\text{T}}_{s}))g_{2}^{*}(y(m{\text{T}}_{s}))$$

where:

- T*_s_* is the sampling period for the *x* and *y* inputs, respectively. In most of the applications both sampling times are identical except for skew and jitter effects ((T_1_ − T_2_) = T_Skew_ + T_Jitter_(*t*)), for simplicity, without loss of generality, skew and jitter can be simulated as well), and
- *g*_1,2_ are defined in Equation (3).

Obviously, the product of a pair of samples of $p_{\left|m-n\right|}=g_{1}(x(n{\text{T}}_{s}))g_{2}^{*}(y(m{\text{T}}_{s})$ in Equation (26) does not follow a Gaussian pdf. Rather, the distribution of *p*_|_*_m_*_−_*_n_*_|_ follows a Rayleigh pdf when the *g* functions are linear (no quantization). In a more general case, having non-linear *g*_1,2_ functions, the distribution of *p*_|_*_m_*_−_*_n_*_|_ follows different trends depending on the quantization scheme. However, the average of many of these products, over a large number of pairs of samples, approaches a Gaussian pdf, as implied by the central limit theorem. The variance of the correlator output can then be calculated as in Equation (27):

(27) $$\sigma_{R_{xy}}^{2}=\langle R_{xy}R_{xy}^{*}\rangle -{\langle R_{xy}\rangle }^{2}$$

where the first term of this addition is expanded as Equation (28):

(28) $$\langle R_{xy}R_{xy}^{*}\rangle =\frac{1}{N_{q}^{2}}\sum_{n=1}^{N_{q}}\sum_{m=1}^{N_{q}}\langle g_{1}(x(n{\text{T}}_{s}))g_{2}^{*}(y(m{\text{T}}_{s}))g_{1}^{*}(x(n{\text{T}}_{s}))g_{2}(y(m{\text{T}}_{s}))\rangle$$

Thence, Equation (28) is split in two parts, the first one takes into account only the elements which have the same *n* and *m* time indexes, and a second part, which is the rest of the Equation (28):

(29) $$\langle R_{xy}R_{xy}^{*}\rangle =\frac{1}{N_{q}^{2}}\left\{\begin{array}{c} \sum_{n=1}^{N_{q}}\langle g_{1}(x(n{\text{T}}_{s}))g_{2}^{*}(y(n{\text{T}}_{s}))g_{1}^{*}(x(n{\text{T}}_{s}))g_{2}(y(n{\text{T}}_{s}))\rangle +\\ \sum_{n=1}^{N_{q}}\sum_{\begin{array}{l} m=1\\ m\neq n \end{array}}^{N_{q}}\langle g_{1}(x(n{\text{T}}_{s}))g_{2}^{*}(y(n{\text{T}}_{s}))g_{1}^{*}(x(mT_{s}))g_{2}(y(m{\text{T}}_{s}))\rangle \end{array}\right\}$$

the first term of Equation (29) can be computed using the properties of the fourth and second statistical moments relationship for zero mean Gaussian random variables [18], as follows:

(30) $$\begin{array}{l} \langle g_{1}(x(n{\text{T}}_{s}))g_{2}^{*}(y(n{\text{T}}_{s}))g_{1}^{*}(x(n{\text{T}}_{s}))g_{2}(y(n{\text{T}}_{s}))\rangle =\\ \;\;\;\;\;\;\;\;\;\;\;\;\;\;\;\;\;\;\;\;\;\;\;\;\;\;\;\;\;\;=\left\{\begin{array}{l} \langle g_{1}(x(n{\text{T}}_{s}))g_{1}^{*}(x(n{\text{T}}_{s}))\rangle \langle g_{2}^{*}(y(n{\text{T}}_{s}))g_{2}(y(n{\text{T}}_{s}))\rangle \\ +\langle g_{1}(x(n{\text{T}}_{s}))g_{2}^{*}(y(n{\text{T}}_{s}))\rangle \langle g_{1}^{*}(x(n{\text{T}}_{s}))g_{2}(y(n{\text{T}}_{s}))\rangle \end{array}\right\}=\\ \;\;\;\;\;\;\;\;\;\;\;\;\;\;\;\;\;\;\;\;\;\;\;\;\;\;\;\;\;=\sigma_{g_{1}(x)}^{2}\sigma_{g_{2}(y)}^{2}+|q[\rho_{xy}(0)]|{\;}^{2}\sigma_{g_{1}(x)}^{2}\sigma_{g_{2}(y)}^{2}=\sigma_{g_{1}(x)}^{2}\sigma_{g_{2}(y)}^{2}\left(1+|q[\rho_{xy}(0)]{|}^{2}\right) \end{array}$$

where:

- $\sigma_{g_{1}(x)}^{2}$ and $\sigma_{g_{2}(y)}^{2}$ are the variances of *g*_1_(*x*) and *g*_2_(*y*), respectively, and
- |*q*[*ρ_xy_*(0)]| is the modulus of the cross-correlation coefficient of the input signals after the non-linear function.

The second term in Equation (30) can be expanded using the same statistical moments relationship:

(31) $$\begin{array}{l} \frac{1}{N_{q}^{2}}\sum_{n=1}^{N_{q}}\sum_{\begin{array}{l} m=1\\ m\neq n \end{array}}^{N_{q}}\langle g_{1}(x(n{\text{T}}_{s}))g_{2}^{*}(y(n{\text{T}}_{s}))g_{1}^{*}(x(m{\text{T}}_{s}))g_{2}(y(m{\text{T}}_{s}))\rangle =\\ \;\;\;\;\;\;\;\;\;\;\;\;\;\;\;\;\;\;\;\;\;\;\;\;\;\;\;\;\;\;\;\;=\left\{\begin{array}{l} \frac{N_{q}-1}{N_{q}^{2}}\sum_{n=1}^{N_{q}}\langle g_{1}(x(n{\text{T}}_{s}))g_{1}^{*}(x(m{\text{T}}_{s}))\rangle \langle g_{2}^{*}(y(n{\text{T}}_{s}))g_{2}(y(m{\text{T}}_{s}))\rangle \\ +\frac{1}{N_{q}^{2}}\sum_{n=1}^{N_{q}}\sum_{\begin{array}{l} m=1\\ m\neq n \end{array}}^{N_{q}}\langle g_{1}(x(n{\text{T}}_{s}))g_{2}^{*}(y(n{\text{T}}_{s}))\rangle \langle g_{1}^{*}(x(m{\text{T}}_{s}))g_{2}(y(m{\text{T}}_{s}))\rangle \end{array}\right\}\\ \;\;\;\;\;\;\;\;\;\;\;\;\;\;\;\;\;\;\;\;\;\;\;\;\;\;\;\;\;\;\;\;=\frac{N_{q}-1}{N_{q}}|q[\rho_{xy}(0)]{|}^{2}\sigma_{g_{1}(x)}^{2}\sigma_{g_{2}(y)}^{2}+\frac{2}{N_{q}^{2}}\sum_{n=1}^{N_{q}}\left(N_{q}-n\right)R_{xx}(n{\text{T}}_{s})R_{yy}(n{\text{T}}_{s}) \end{array}$$

where:

- *R_xx_*(*n*T*_s_*) and *R_yy_*(*n*T*_s_*) are the auto-correlation of the *g*_1_(*x*) and *g*_2_(*y*), respectively taking into account all the samples between sample 1 and the *N_q_*, as in Equation (12).

The first part of the variance of the correlation is obtained by combining the results obtained in Equations (30) and (31):

(32) $$\begin{array}{l} \langle R_{xy}R_{xy}^{*}\rangle =\left\{\begin{array}{c} \frac{\sigma_{g_{1}(x)}^{2}\sigma_{g_{2}(y)}^{2}}{N_{q}}\left(1+|q[\rho_{xy}(0)]{|}^{2}\right)+\\ \frac{N_{q}-1}{N_{q}}|q[\rho_{xy}(0)]{|}^{2}\sigma_{g_{1}(x)}^{2}\sigma_{g_{2}(y)}^{2}+\frac{2}{N_{q}^{2}}\sum_{n=1}^{N_{q}}\left(N_{q}-n)R_{xx}(n{\text{T}}_{s})R_{yy}(n{\text{T}}_{s}\right) \end{array}\right\}\\ \;\;\;\;\;\;\;\;\;\;\;\;\;\;\;\;\;\;\;\;\;\;\;\;\;\;=\frac{\sigma_{g_{1}(x)}^{2}\sigma_{g_{2}(y)}^{2}}{N_{q}}+\frac{2}{N_{q}^{2}}\sum_{n=1}^{N_{q}}(N_{q}-n)R_{xx}(n{\text{T}}_{s})R_{yy}(n{\text{T}}_{s})+|q[\rho_{xy}(0)]{|}^{2}\sigma_{g_{1}(x)}^{2}\sigma_{g_{2}(y).}^{2} \end{array}$$

Finally, the value of the variance of the *N_q_*-samples averaged correlation is given by:

(33) $$\sigma_{R_{xy}}^{2}=\frac{\sigma_{g_{1}(x)}^{2}\sigma_{g_{2}(y)}^{2}}{N_{q}}+\frac{2}{N_{q}^{2}}\sum_{n=1}^{N_{q}}\left(N_{q}-n)R_{xx}(n{\text{T}}_{s})R_{yy}(n{\text{T}}_{s}\right)$$

and arranging Equation (33), the variance appears in its usual form, as it is known in the literature [17]:

(34) $$\sigma_{R_{xy}}^{2}=\frac{\sigma_{p}^{2}}{N_{q}}+\frac{2}{N_{q}^{2}}\sum_{n=1}^{N_{q}}\left(N_{q}-n)R_{xx}(n{\text{T}}_{s})R_{yy}(n{\text{T}}_{s}\right)$$

where, $\sigma_{p}^{2}=\sigma_{g_{1}(x)}^{2}\sigma_{g_{2}(y)}^{2}$ is the variance for each sample of the correlation (*N_q_* = 1).

**Figure 12.**
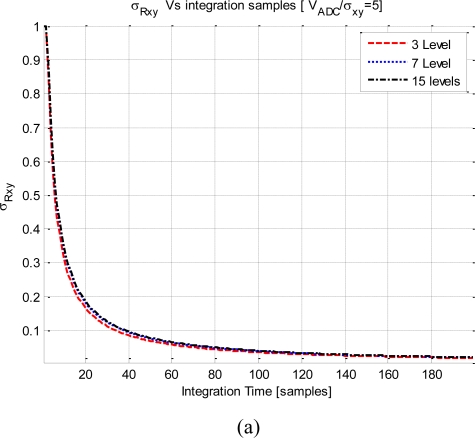

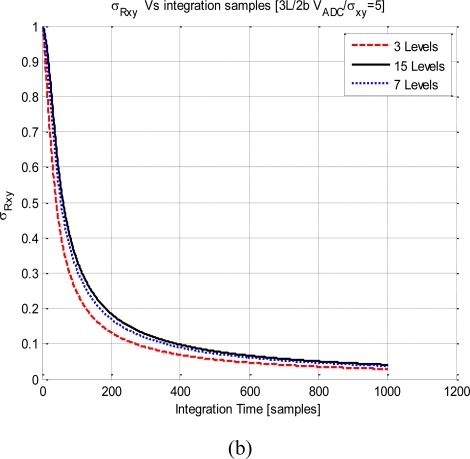
Impact of the number of averaged samples on the $\sigma_{R_{xy}}^{2}$, **(a)** shows the impact for a 3 level quantization scheme and different sampling rate, and **(b)** shows a constant sampling rate changing the number of quantization levels.

Following Equation (25), Figure 12 shows the evolution of $\sigma_{R_{xy}}^{2}$ with the integration time. The signals considered in these plots have a noise equivalent squared bandwidth of pass-band of 2*B*, *ρ_xy_*(0) = 1, and V_ADC_ = 5*σ*_x,y_.

Figure 12(a) shows the results for the three quantization levels, all the curves converge to zero asymptotically. The *F_s_* = 0.75F_Nyquist_ curve converges faster, because its samples are uncorrelated among them. For a given number of averaged samples, the variance of the quantized correlation increases with the sampling rate, because successive samples are more and more correlated. Recall that, abscises axis of these plots are displayed in number of averaged samples, not in integration time units. Obviously, for a given integration time, the plot with the highest sample rate has the lowest $\sigma_{R_{xy}}^{2}$, it has had time to average more samples despite that for a given number of samples it exhibits the worst performance.

In Figure 12(b) the sampling rate has been frozen to *F_s_* = F_Nyquist_ and two quantization schemes has been analyzed (3 and 15 levels). The three-level curve converges more quickly to zero than the 15 levels curve. This is because the fringe washing function is shaper due to the higher non-linear distortion in three levels than in the 15 levels quantization. This effect is equivalent to have more uncorrelated samples. Despite the mean of the correlation is lower for the three-level that the 15-level quantization, the variance of the correlation is also lower in this case.

## 8. Conclusions

The impact of a general quantization scheme has been expressed and analyzed as a non-linear transformation. Despite the quantization can significantly affect the value of the ideal cross-correlation (*ρ_xy_*), it is possible to recover the ideal correlation (*ρ_xy_*) from the measured one (*R_xy_*) using then the function *ρ_xy_* = *q*^−1^[*R_xy_*], which can be always numerically obtained. It has been found that for each quantization scheme there is an optimum configuration of the V_ADC_/*σ_x,y_* relationship for which the linearity with respect the ideal correlation is minimized. Furthermore, quantization distorts and spreads the cross-correlation spectrum, decreasing the radiometric resolution.

Sampling also has an impact in the spectrum of the cross-correlation function, and depending on the sampling rate, replicas of the spectrum can overlap the main spectrum. This can be due to a sampling rate below the Nyquist criterion or, even above the Nyquist criterion, due to the non-linear quantization process which spreads the spectrum. In both cases, there is an impact on the SNR, and so on the radiometric resolution. Other sampling effects have been analyzed such as the clock inaccuracies: skew and jitter.

Finally, the impact of quantization and sampling on the variance of the measurements has been studied. It has been found that it strongly depends on the relationship between the bandwidth of the cross-correlation and the sampling rate. As the sampling rate increases, the successive samples are more correlated, so they add less new information to the measurements and the variance decreases slowly.

## Figures and Tables

**Table 1.** Summary of the critical design parameter VADC/σx,y. Relationship between its optimal configuration and the minimum root mean square error obtained for some quantization schemes.

**Levels**	**V_ADC_/σ_x,y_ optimum**	**RMSE minimum [%]**
2 (1 bit)	no optimum	14.2
3 (1.6 bits)	0.7	4.7
7 (2.8 bits)	1.8	1.4
15 (3.90 bits)	2.2	0.3
31 (4.95 bits)	3.0	0.02
63 (5.97 bits)	3.5	0.0066

**Table 2.** Summary of the spectrum distortion and spread for V_ADC_ = 5σ*_x,y_* and different quantization schemes.

**Levels**	**Pass-band**	**Rejected band increase**
2 (1 bit)	80%	13%
7 (2.8 bits)	87%	5.0%
15 (3.90 bits)	97%	1.3%
31 (4.95 bits)	99%	1.0%
Ideal	100%	0.0%
